# 3D/2D Bilayerd Perovskite Solar Cells with an Enhanced Stability and Performance

**DOI:** 10.3390/ma13173868

**Published:** 2020-09-01

**Authors:** Hyeon-Seo Choi, Hui-Seon Kim

**Affiliations:** Department of Chemistry and Chemical Engineering, Inha University, Incheon 22212, Korea; hs_choi@inha.edu

**Keywords:** perovskite, solar cell, 3D/2D, stability, efficiency

## Abstract

The formation of a thin 2D perovskite layer on the surface of 3D perovskite films has become a popular strategy for obtaining a high-efficiency perovskite solar cell (PSC) with an ensured device stability. In this review paper, various experimental methods used for growth of the 2D layer are introduced with the resulting film properties. Furthermore, a variety of organic cation sources for the 2D layer, ranging from alkyl to phenyl ammonium, are explored to investigate their impact on the device stability and photovoltaic performance.

## 1. Introduction

Since the advent of solid-state perovskite solar cells (PSCs) in 2012 [1,2], intensive research has been conducted to exploit the outstanding optoelectronic properties of perovskite material, such as the ambipolar charge-transfer [3], balanced long-range carrier diffusion lengths [4], low defect density with a shallow distribution [5], and high defect tolerance [6], which is particularly favorable for obtaining a low temperature processability and thus has the benefit of producing cost-effective technology based on the solution process at a low temperature [7]. The certified record efficiency of PSC was shown to be 25.2% in 2020 [8], which makes PSC one of the most promising technologies for the photovoltaic market. Nevertheless, PSCs still face the long-term stability issue, which needs to be overcome for commercialization. The phase instability problem is inherent in perovskite material, and is closely related to the low activation energy of ion migration [9,10]. The ease of vacancy formation and migration encourages the crystal lattice of the perovskite to collapse and convert to lead iodide or/and the energetically favored non-perovskite phase under various environmental stresses of photon, moisture, thermal, and voltage bias.

**Figure 1:**
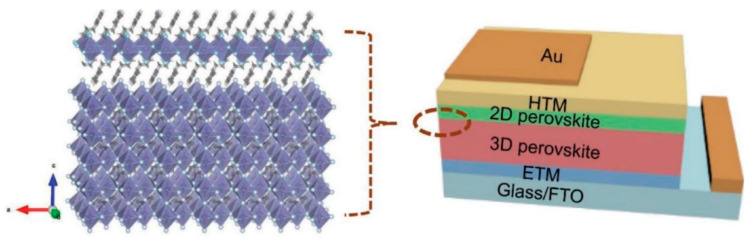
Illustration of the 3D/2D bilayered perovskite structure and perovskite solar cell (PSC) device. Reprinted with permission from Ref. [28] Copyright 2018 Wiley-VCH.

Not only to remedy these instabilities, but also to enhance the performance, diverse attempts have been made, such as compositional engineering using mixed-cations and -halides [11,12,13] and the employment of an interlayer between the perovskite and selective contacts [14,15,16]. Meanwhile, 2D perovskite, where bulky cations invade 3D perovskite and slice the lattice parallel to the substrate, has attracted attention owing to its superior moisture resistance due to the hydrophobicity of spacer cations [17,18]. Mohite et al. developed the quasi-2D perovskite system based on methyl ammonium iodide (MAI) and butyl ammonium iodide (BAI), leading to (BA)_2_(MA)_2_Pb_3_I_10_ and (BA)_2_(MA)_3_Pb_4_I_13_, which showed considerably improved light- and moisture-stabilities compared to 3D perovskite (MAPbI_3_) [19]. In spite of the notable stability enhancement, the power conversion efficiency (PCE) of PSCs based on quasi-2D perovskite is generally lower than that of 3D-based PSCs [18,19,20], which is attributed to the inferior optoelectronic properties of 2D perovskite, and similarly to the insulating characteristics [19,21]. The limitation of 2D or quasi-2D perovskite was recently resolved by introducing 3D/2D hybrid perovskite, where each advantage of 3D and 2D perovskite was comprehensively employed to ensure a superb performance in terms of both the stability and PCE [22,23]. In 2016, Shi et al. conceived the hybrid structure, as illustrated in Figure 1, where an extremely thin 2D layer is deposited on the top of the 3D bulk film, leading to a remarkable moisture stability and a PCE of 13.86% with an increased open-circuit voltage (*V*_OC_) [24]. The promising results encouraged the 3D/2D hybrid perovskite system to be intensively explored with many different bulky cations in a variety of forms [25,26,27], which is now regarded as an effective strategy for assuring the stability without compromising on the photovoltaic performance [27].

In this review paper, we focus on 3D/2D hybrid perovskite-based PSCs, where the 2D perovskite is deposited on the 3D bulk film in the form of an extremely thin layer. The 3D/2D bilayered-perovskite is thoroughly investigated from the perspectives of the experimental process, resultant morphology, organic salt type used for the 2D layer, and effect on the performance and the stability.

## 2. Process

### 2.1. In-Situ Growth Method

**Figure 2:**
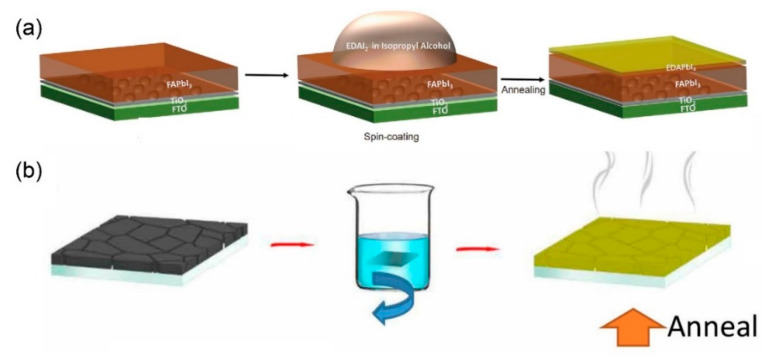
Schematic experimental process of the in-situ method conducted by (**a**) spinning and (**b**) dipping. Reprinted with permission from ref 29 Copyright 2019 Springer Nature and Ref. [25] Copyright 2019 American Association for the Advancement of Science (AAAS).

The in-situ growth of 2D perovskite is the most widely used process for forming the 3D/2D bilayered perovskite structure, since it is easily implemented by a few additional steps after routine preparation of the 3D bulk film. Figure 2 shows a schematic experimental process of the in-situ growth of a 2D capping layer, which can be adapted with either spinning (Figure 2a) or dipping (Figure 2b) to form a 2D thin layer on top of a 3D perovskite film [25,29]. Organic salt, composed of bulky organic cations and counter halide (see Section 4. Organic salt for 2D perovskite for details), is dissolved in isopropyl alcohol (IPA) [24,25,26,28,29,30,31,32,33,34,35,36,37,38,39,40,41] or chlorobenzene (CB) [36,42] and dripped on the surface of a spinning 3D perovskite film, whose excess PbI_2_ readily reacts with the organic salt, leading to the formation of a 2D capping layer on the surface during the post annealing process (Figure 2a). Alternatively, an immersion of the 3D perovskite film in the IPA solution containing the organic salt for 2D can be adopted, instead of dripping the solution, in order to induce the 2D capping layer (Figure 2b) [25]. The thickness of the 2D layer can be controlled by the concentration of the solution containing the organic salt [24]. It has been found that additional flash annealing of the 3D perovskite film at a high temperature facilitates the formation of the 2D perovskite layer by exposing the Pb-I framework of the 3D structure. For flash annealing, MAPbI_3_ bulk film is reversely placed on a hot plate with a gap of 3 mm at 300 °C for less than 10 s to release MAI on the surface. The exposed Pb-I framework is reacted with 5-aminovaleric acid iodide (AVAI), resulting in (AVA)_2_PbI_4_ with an improved interface [26]. The in-situ growth method for the 2D capping layer can be further extended to form double 2D passivation layers on the top and bottom of the 3D perovskite film [41].

Notably, a salt mixture of 3D and 2D is used in the in-situ growth method to form a 3D/quasi-2D layered structure. An IPA solution containing BAI and formamidinium iodide (FAI) with different ratios was employed in an attempt to form the capping layer on top of the 3D perovskite with excess PbI_2_, resulting in the highest photovoltaic performance when the same molar ratio between BAI and FAI was employed (BAI:FAI = 1:1) to form a quasi-2D layer—(BA)_2_(FA)_n−1_Pb_n_I_3n+1_—as confirmed by x-ray diffraction (XRD) [39]. Similarly, penylethylammonium (PEA) was blended with FA at the same molar ratio, which led to a capping layer composed of a double phase comprised of (PEA)_2_PbI_4_ and (PEA)_2_(FA)Pb_2_I_7_ [33].

### 2.2. Anti-Solvent Method

**Figure 3:**
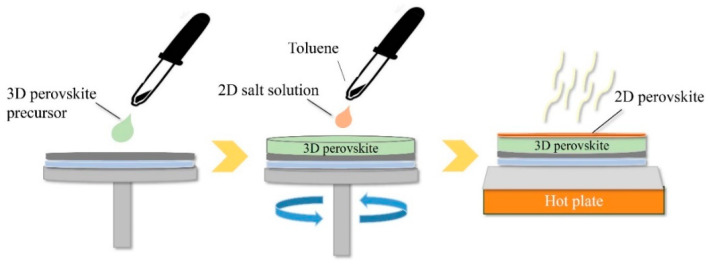
Schematic experimental process of the anti-solvent method.

The anti-solvent method has been well-established as a conventional recipe for obtaining a high-quality 3D perovskite film [43]. The anti-solvent (e.g., CB, toluene, etc.) is employed as a second solvent to induce quick nucleation for fast crystallization of the 3D film [43]. When the anti-solvent is used as a solvent for the 2D organic salt, the in-situ 2D growth method can be practically combined with the conventional anti-solvent method, as illustrated in Figure 3. The anti-solvent containing the organic salt for 2D is directly dripped on the spinning substrate, which is wet with a precursor solution of the 3D perovskite. The dripping process of the anti-solvent is followed by a one-step annealing process to form both 3D and 2D perovskite layers at the same time. Toluene containing phenylethylammonium iodide (PEAI) was used as the anti-solvent for a 3D/2D bilayered perovskite structure [44]. A successful deposition of the 2D capping layer was subsequently verified by the time-of-flight secondary ion mass spectroscopy (TOF-SIMS) depth profile, where the PEA cation for the 2D capping layer was monitored across the 3D film by the anti-solvent method, but specifically concentrated near the surface with a sharp increase in the intensity, indicating the formation of a 2D thin layer on the surface.

### 2.3. Solvent-Free Method

A method with the absence of solvent has emerged to form the 2D capping layer. The aforementioned in-situ growth and anti-solvent methods are based on the solution process, where treatment with the solution containing a salt for 2D is likely to affect the priorly formed-3D perovskite film. In addition, the solution-based methods are not only difficult to use to control the 2D distribution in 3D, but also troublesome in terms of avoiding the impact of solvent (IPA) [22,38]. To this end, the solvent-free method has been tested by using vapors to form a thin 2D layer on the surface of the 3D film in various conditions. In the vapor deposition or vapor-assisted method, the 3D/2D bilayered structure is developed by the permeation of organoamine gases for 2D into the 3D film from the surface [45]. The 3D perovskite film is exposed to the vapors being generated from the heated organoamine liquid or organic salt [46]. Chen et al. formed a 2D perovskite capping layer by exposing the 3D perovskite film, prepared by the conventional solution method, to butylamine vapor in a sealed box containing an open bottle filled with butylamine liquid, leading to a surface conversion from 3D to 2D perovskite [45]. Meanwhile, Xu et al. built an entire 3D/2D perovskite structure by vapor deposition [46]. The PbI_2_ was deposited on a TiO_2_ substrate by a thermal vacuum evaporator, which was followed by the vapor deposition of MAI powder in a vacuum oven at 180 °C for 30 min to convert PbI_2_ to MAPbI_3_. The prepared 3D bulk film, MAPbI_3_, was sequentially exposed to BAI vapor in the vacuum oven at 120 °C, with the exposure time being varied from 5 to 60 min to form the 2D layer on the top. It was found that the quality and conversion degree of the 2D layer are fully controllable by modulating the vapor-treatment time in solvent-free methods [45,46]. Similarly, 3D and 2D perovskite layers can be independently deposited by using a thermal vacuum evaporator. Bolink et al. carried out the whole vapor disposition process in a thermal evaporator, where the deposition rate and the expected deposition thickness of each source were closely monitored by quartz crystal microbalance, adjusting the temperature for sublimation [47]. The 3D bulk film was obtained by the coevaporation of PbI_2_ and MAI. The 2D capping layer was sequentially deposited on top of MAPbI_3_ by another coevaporation process of PbI_2_ and PEAI, which resulted in the 3D/2D bilayered structure. Thanks to the benefit of the vacuum process, a more complicated architecture composed of 2D/3D/2D was further enabled by the evaporator, leading to a neat interface between 2D and 3D layers [47].

### 2.4. Other Methods

A 3D/2D graded structure can be also prepared with a single perovskite precursor solution containing salts for both 3D and 2D. A salt for 2D—(CF_3_)_3_CO(CH_2_)_3_NH_3_I—was blended with FAI, MAI, and PbI_2_ in DMSO [34]. The prepared solution was spin-coated on the substrate with the conventional anti-solvent and annealed to remove the residual solvent, leading to a graded film where photoluminescence (PL) spectra of the top side were different to those of the bottom side. The excitation of the bottom (substrate) side showed a single peak indicative of the 3D bulk film, while the excitation of the other (top) side demonstrated additional peaks corresponding to the 2D perovskite, coupled with the peak for 3D. It was noted that the fluorine-rich cation grants a self-assembly tendency and orthogonal peculiarity, which would be responsible for the spontaneously formed 2D perovskite as a thin capping layer on top of the 3D perovskite [34,48].

## 3. Morphology

**Figure 4:**
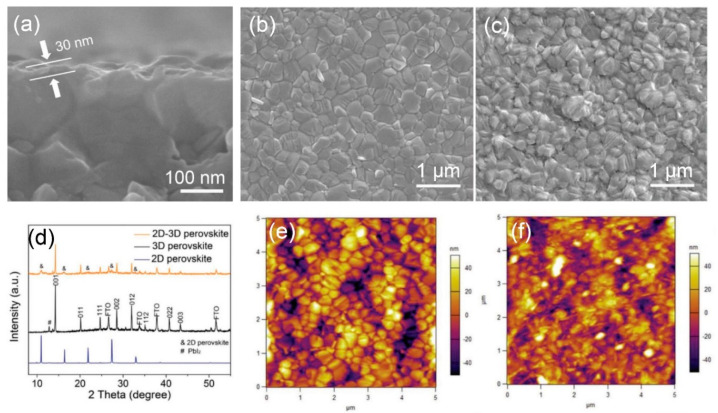
General morphology changes caused by the in-situ growth of the 2D capping layer. (**a**) Cross-sectional scanning electron microscope (SEM) image of the 3D/2D bilayered perovskite structure. Surface SEM images of (**b**) the 3D perovskite film and (**c**) 3D/2D bilayered structure with the in-situ-grown 2D capping layer. (**d**) X-ray diffraction (XRD) patterns of the respective structure. Atomic force microscope (AFM) images of (**e**) the 3D perovskite film and (**f**) 3D/2D bilayered structure with the in-situ-grown 2D capping layer. Reprinted with permission from Ref. [28] Copyright 2018 Wiley-VCH.

When a 3D/2D bilayered structure is formed, the morphology of the resultant heterostructure is considerably distinguished from that of a single 3D or 2D structure. The scanning electron microscope (SEM) images in Figure 4a–c represent the morphology change caused by the in-situ growth of the 2D perovskite (PEA_2_PbI_4_) layer on the 3D (α-FAPbI_3_) film. Figure 4a shows the cross-sectional morphology of 3D/2D bilayered structures where a 30 nm-thick 2D capping layer (generally ranging from 9 mm [25] to 100 nm [31]) is grown on the surface of the 3D bulk film. Figure 4b shows a surface image of the compact 3D film, where the white flake at grain boundaries indicates the excess PbI_2_ exposed on the 3D surface [24,29,37,39]. On the other hand, a distinctive surface morphology is observed by the in-situ-grown 2D capping layer, which fully covers the 3D film (Figure 4c). It is notable that the PbI_2_ flake on the 3D surface disappears in the 3D/2D bilayered structure due to the reaction with salts during the in-situ growth of the 2D capping layer [24,28,29,37,39]. XRD patterns also ensure a reaction between the excess PbI_2_ of the α-FAPbI_3_ film with permeated PEAI salts during the in-situ growth (Figure 4d), showing a negligible PbI_2_ peak at 12.7° in the 3D/2D bilayered structure. Accordingly, the 3D/2D bilayered structure displays hybrid patterns dominantly governed by the 3D α-FAPbI_3_ bulk film, with weak patterns for the 2D PEA_2_PbI_4_ capping layer. Importantly, the improved surface morphology with a reduced roughness is one of the general features observed from the 3D/2D bilayered structure [24,25,26,36,39]. Figure 4e,f show atomic force microscope (AFM) images of the 3D and 3D/2D bilayered structure, respectively. The formation of the 2D capping layer results in a smoother surface with a root-mean square (RMS) of 17.8 nm, while the 3D film demonstrates an RMS of 20.8 nm.

**Figure 5:**
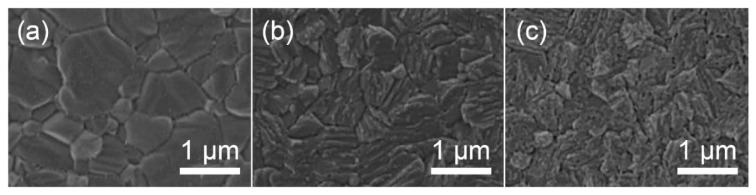
Morphology change when increasing the exposure time to vapor. SEM images of as-prepared (**a**) 3D perovskite film and the films with butyl ammonium iodide (BAI) vapor treatment for (**b**) 5 and (**c**) 60 min. Reprinted with permission from Ref. [46] Copyright 2019 Elsevier Ltd.

The morphology of the 3D/2D bilayered structure is found to be significantly altered by varying the reaction time for the growth of the 2D layer. Figure 5 shows a change in the film morphology as a function of the vapor exposure time in the solvent-free method [46]. The surface morphology turns into platelet-like crystals due to the layered structure (2D) grown on the surface when increasing the exposure time to BAI vapor. As the formation of the 2D layer is more facilitated after a full coverage of the 3D surface, the roughness is reversely increased due to the pronounced impact of the 2D morphology [46]. The surface morphology of the 3D/2D bilayered structure occasionally leads to a decreased grain size, compared to the 3D perovskite, which is also attributed to the crystal morphology of the 2D layer grown on the surface, without affecting the underlying 3D bulk film [44,46].

**Figure 6:**
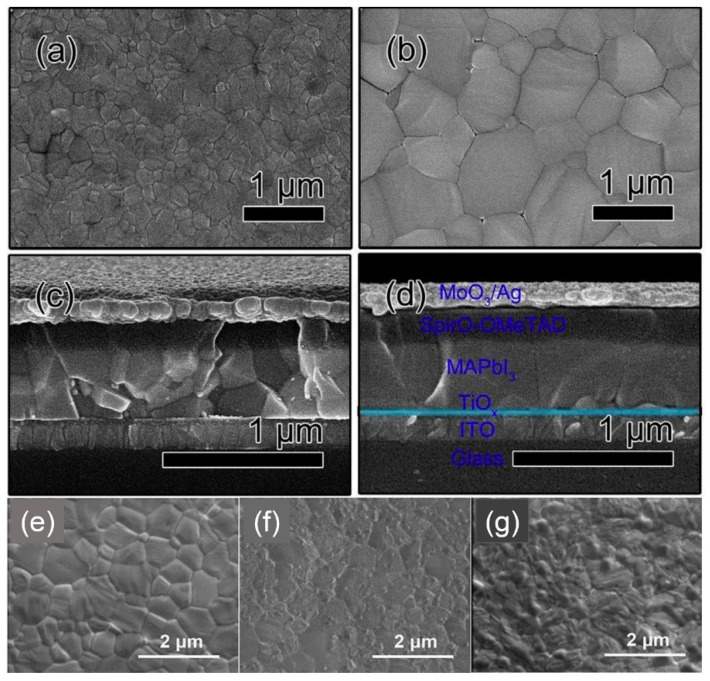
Effect of the precursor on the morphology change of the in-situ-grown 2D layer. Surface SEM images of (**a**) 3D perovskite and (**b**) a 3D/2D bilayered structure induced by diethylammonium bromide (DABr). Cross-sectional SEM images of devices employing (**c**) 3D perovskite and (**d**) a 3D/2D bilayered structure induced by DABr. Reprinted with permission from Ref. [30] Copyright 2019 Elsevier. Surface SEM images of (**e**) 3D perovskite film and a 3D/2D bilayered film caused by a post-solution treatment with (**f**) benzylamine (BA) and (**g**) BAI. Reprinted with permission from Ref. [36] Copyright 2018 American Chemical Society.

Furthermore, the morphology change is highly dependent on the precursor source used for the 2D layer. While the precursor employed for the 2D layer is mostly adopted as a form of iodide or amine species, diethylammonium bromide (DABr) was introduced as a salt for the in-situ growth of the 2D layer [30]. Post-treatment with DABr not only formed a 2D capping layer, but also induced a secondary growth of the 3D film. Figure 6a,c present the surface and cross-sectional SEM images of the pristine 3D bulk film. Growth of the 2D layer was observed when using 3 mg/mL of DABr solution, which evolved into Ostwald ripening of the underlying 3D MAPbI_3_ film when increasing the concentration of DABr solution. An apparent increase in grain size can be observed in Figure 6b for when Br^−^ was incorporated by using 5 mg/mL DABr solution. A cross-sectional image of the 3D/2D bilayered structure based on 5 mg/mL DABr shown in Figure 6d confirms that post-treatment with DABr considerably enhanced the crystallinity of the underlying 3D MAPbI_3_ film, as evidenced by the increased grain size. On the other hand, a different tendency of the 2D capping layer can be observed with respect to the precursor source containing the same organic species. n-butylamine (n-BA) and n-BAI were used as precursors for the 2D layer, where BA and BAI were dissolved in CB and IPA, respectively [36]. Figure 6e shows the conventional surface morphology of the pristine 3D bulk layer. The post-solution treatment with BA (Figure 6f) and BAI (Figure 6e) considerably altered the pristine morphology due to the formation a 2D capping layer, as proposed in the following equations [36]:

2BA + MAI + MAPbI_3_ → (BA)_2_PbI_4_ + 2MA ↑, (1)

2BAI + *n*MAPbI_3_ ↔ (BA)_2_(MA)*_n_*_−__1_Pb*_n_*I_3_*_n_*_+__1_ + MAI. (2)

It was found that the resultant morphology induced by BA solution is analogous to the surface obtained by the post-treatment with amine gas, which readily dissolves the perovskite surface and reforms the surface by the removal of amine gas due to its evaporation [49]. Therefore, the formation of amine gas (MA) during the in-situ growth by BA solution, as suggested in Equation (1), is likely to reconstruct the surface and thus result in a smoother surface compared to BAI solution (Figure 6f). On the other hand, common 2D layer formation is expected from the in-situ growth caused by BAI solution, as shown in Equation (2), leading to a relatively rougher surface than the case of BA (Figure 6e).

Sometimes, a small morphology change is observed after the growth of a 2D layer, where an extremely thin 2D layer is piled up without causing any major change in the surface morphology [34,45].

## 4. Organic Salt for 2D Perovskite

Halide perovskite has an ABX_3_ crystal structure, where a BX_6_ corner-sharing cage surrounds the A cation (A = FA^+^, MA^+^, or Cs^+^; B = Pb^2+^ or Sn^2+^; and X = Br^−^ or I^−^). In order for the ABX_3_ structure to remain stable, respective ions have a certain range of radii and balance with other ions. The formation possibility of the perovskite structure can be predicted by the Goldschmidt tolerance factor (*t*) in Equation (3), where *r_A_*, *r_B_*, and *r_X_* denote the ionic radii of A, B, and X, respectively [50].

(3) $$t=\;\frac{r_{A}\;+\;r_{X}}{\sqrt{2}\;\left(r_{B}+\;r_{X}\right)}$$

A stable crystal structure of 3D perovskite is expected when *t* ranges between 0.8 and 0.9 [51]. While FA^+^, MA^+^, and Cs^+^ meet the tolerance range for a 3D structure with given ionic radii of Pb^2+^ and I^−^, other larger species of organic cations in the A site can result in a distortion and slice the 3D lattice in parallel, resulting in a low dimensional structure of 2D [50]. A variety of organic salts with a larger size have been employed to grow the 2D layer for the 3D/2D bilayered perovskite. In Table 1, organic salts employed for the 2D capping layer are listed with corresponding experimental methods and resulting photovoltaic parameters of the 3D/2D bilayered structure compared to the pristine 3D film.

Before the active adoption of a 3D/2D bilayered structure in PSCs, ammonium salts with a bulky phenyl alkyl chain (ex. PEA^+^) were introduced to PSC as a form of 2D layer [18]. The (PEA)_2_(MA)_2_Pb_3_I_10_-based layered perovskite showed a promising device stability due to an increased moisture resistance, in spite of its poor performance. Henceforward, ammonium salt with a long alkyl chain (ex. BA^+^) was also applied for quasi-2D perovskite to boost the photovoltaic performance with an ensured stability of the perovskite layer [17]. It was noted that the improved stability caused by the 2D structure tended to scarify the efficiency by avoiding the 3D structure, which was resolved by introducing the 3D/2D bilayered structure based on cyclopropyl ammonium as a cation for the 2D capping layer [24]. Since the first introduction of the 3D/2D structure, various cation sources have been consistently explored, in order to find the most effective 2D capping layer grown on the 3D surface [31,33,36,37]. A longer alkyl chain not only increases the stability, but also improves the interface, showing a reduced number of surface defects, as evidenced by the prolonged PL life time and reduced trap density [31,37]. Therefore, BA^+^ and PEA^+^ became the major cations for the dense growth of the 2D layer thanks to the superior stability, without compromising the efficiency [18,19]. On the other hand, attempts to employ a fluorinated compound in perovskite were made in various ways, including perovskite compositional engineering, as well as surface passivation with fluorinated species [52,53]. The fluorinated species recently evolved to sources for the 2D layer to take advantage of their remarkable properties, such as their strong hydrophobicity, lipophobicity, and self-organization [48]. Since the fluorous cation (A43) had an outstanding effect on both the stability and efficiency [34], intensive efforts have been made to investigate other fluorinated species, which are still regarded as the most promising cation sources for the 2D capping layer, showing good examples of FPEA^+^ and FEA^+^ [25,40]. Thiophene species were also studied as sources for 2D layer formation, displaying an enhanced stability due to the hydrophobicity of the aromatic cation [54]. Among the explored thiophene family, 2-TEA showed the highest effect by forming a robust 2D capping layer, being ascribed to the relatively longer chain of ethyl than that of methyl [35]. Most of the sources employed for the 2D capping layer are in the form of (phenyl)alkyl-amine or -ammonium halide salt. During the growth of the 2D layer on the top of the 3D film, the positive ammonium functional group of the cation source for 2D can passivate the cationic vacancies exposed at the 3D surface, whereas the halide, mostly iodide, supplied as a counter part of the cation source for 2D is prone to passivate the anionic vacancies on the surface of the 3D film [31,55]. Furthermore, the carboxyl group of AVA-5 enables the formation of a uniform 2D capping layer by the aid of hydrogen bonding with halogen of underlying 3D perovskite film [26].

## 5. Beneficial Effects Obtained by Employing 3D/2D Bilayered Perovskite

While the conventional 3D perovskite structure exhibits a low stability, particularly in terms of moisture resistance, the low-dimensional 2D layered structure demonstrates an outstanding resistance to various environmental stresses, including light, heat, and moisture [17,18,56,57]. The establishment of the 3D/2D bilayered structure not only ensures the structural stability of the perovskite crystal phase, but also increases the photovoltaic performance of PSCs, mainly due to the suppressed recombination caused by the passivation effect.

### 5.1. Enhanced Stability

#### 5.1.1. Long-Term Stability

**Figure 7:**
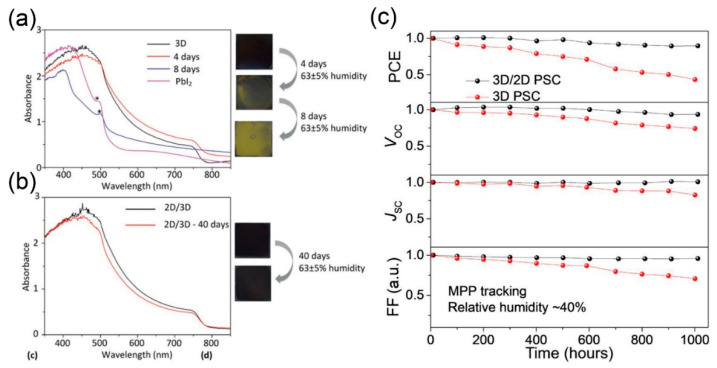
Effect of 3D/2D bilayered perovskite on the device’s long-term stability. Absorbance spectra and photo images of (**a**) 3D and (**b**) 3D/2D bilayered perovskite film as a function of aging time. Reprinted with permission from ref. 24 Copyright 2016 The Royal Society of Chemistry. (**c**) Long-term photovoltaic parameters of un-capsulated PSCs monitored under one sun illumination with a humidity (RH) of ~40% at maximum power point tracking. Reprinted with permission from Ref. [25] Copyright 2019 AAAS.

Under high humidity of 63 ± 5% in ambient air at room temperature (RT), the pristine 3D perovskite film easily turned yellow, with decreased absorbance at the perovskite absorption window (ca. 400–770 nm), which is indicative of the decomposition of the 3D perovskite structure leaving yellow PbI_2_ (Figure 7a) [24]. It is notable that the absorbance peak corresponding to PbI_2_ (~540 nm, denoted as *) was obviously shown after 8 days for full degradation of the 3D structure, while the 3D/2D bilayered structure retained a black phase without any development of the PbI_2_ peak at ~540 nm, even after 40 days under the same condition (Figure 7b) [24]. The robust 3D/2D bilayered structure also affects the device stability, leading to a remarkably improved long-term stability. In inert atmosphere (Ar), a PSC based on 3D/2D maintained 90% of the initial PCE after 32 days, while the one with 3D retained 67% of the initial PCE [26]. Furthermore, the shelf stability of PSCs is also highly dependent on the inclusion of a 2D capping layer. The 3D/2D bilayered-based device, stored in the dark with humidity (RH > 50%), retained 86% of the initial PCE for 100 h, while the 3D-based one dropped to 61% of the initial PCE due to overall decreases in every photovoltaic parameter, including short-circuit photocurrent density (*J*_SC)_), *V*_OC_, and fill factor (FF) [31]. Figure 7c shows a remarkable effect of the 3D/fluorinated-2D bilayered structure in a harsh condition, where the devices were exposed to 1 sun illumination with a relative humidity (RH) of around 40% and kept at the maximum power point (bias voltage stress) during the measurements. In total, 90% of the initial PCE of the 3D/2D-based device was noticeably maintained for 1000 h, even in the intensified stress environments with consistent photon-, moisture- and voltage-bias.

#### 5.1.2. Moisture Stability

**Figure 8:**
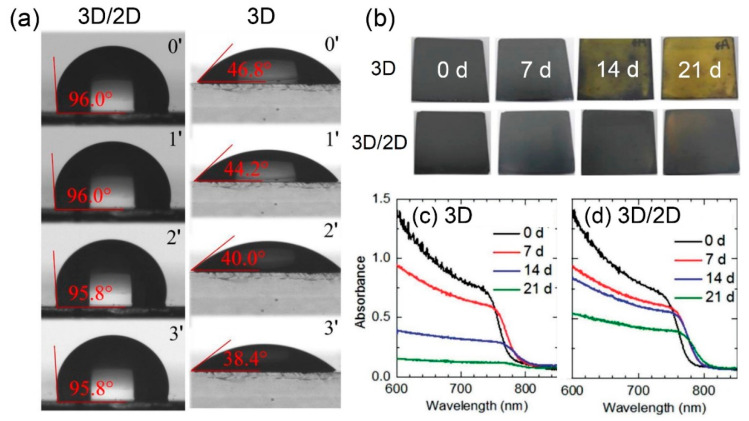
Effect of the 3D/2D bilayered structure on the moisture resistance. (**a**) Contact angle of the water droplet contact angle on top of 3D/2D (left) and 3D (right) as a function of the loading time. Reprinted with permission from Ref. [25] Copyright 2019 AAAS. (**b**) Optical color changes of 3D (top) and 3D/2D (bottom) films stored in a closed vessel with 85 ± 10%. UV-Vis absorption spectra of (**c**) 3D and (**d**) 3D/2D films stored in a closed vessel with 85 ± 10%. Reprinted with permission from Ref. [32] Copyright 2018 WILEY-VCH.

Moisture is one of the major causes of the degradation of 3D perovskite. H_2_O diffuses into 3D bulk film with a low hydrophobicity, consequently leading to a hydrate form, which is photo-inactive [58,59]. 2D perovskite, however, exhibits a superior hydrophobicity, since large aliphatic or aromatic cations are employed [18,57]. Therefore, 3D/2D bilayered perovskite exhibits a great moisture stability by adopting the hydrophobic surface of the 2D capping layer and thus effectively blocks the permeation of water. Figure 8a represents contact angles of water droplets on the surface of a 3D/2D bilayered structure and pristine 3D as a function of the droplet loading time. The 3D/2D bilayered perovskite resulted in a much higher contact angle of 96.0°, compared to 46.8° for the pristine 3D film. While the contact angle of the 3D film gradually decreased when increasing the loading time, that of the 3D/2D bilayered structure was mostly maintained, indicating greatly improved moisture tolerance by the 2D capping layer [25]. As expected from the contact angle experiments, the 3D/2D bilayered structure effectively protects the underlying 3D perovskite. Figure 8b–d indicate the moisture resistance of perovskite films in a closed vessel with RH of 85 ± 10% at RT. The black 3D film turned yellow after 14 days, which was considerably retarded in the 3D/2D bilayered structure (Figure 8b). The observed tendency was confirmed by absorbance spectra, where a rapid decrease in the absorbance of 3D film (Figure 8c) was delayed in the 3D/2D structure owing to the presence of a 2D layer on the 3D film (Figure 8d). An XRD study also verified the high moisture tolerance of 3D/2D perovskite films [32]. When 3D film was kept under RH of 80%, decomposition of the PSC led to the appearance of new XRD peaks at 28.45° corresponding to PbO, 8.35° and 10.31° for (CH_3_NH_3_)PbI_3_∙H_2_O, and 39.36° for AgI [44]. Meanwhile, the 3D/2D-based PSC remained stable, without new peaks, demonstrating that the hydrophobic terminals of the 2D capping layer effectively blocked H_2_O invasion.

#### 5.1.3. Thermal Stability

**Figure 9:**
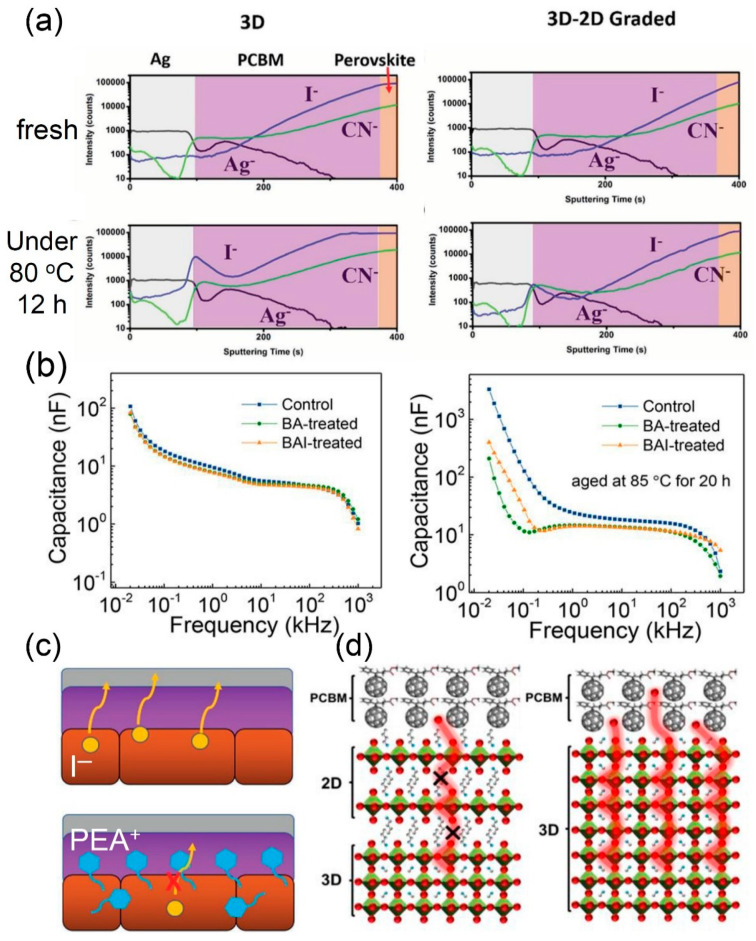
Thermal-induced ion migration in perovskite films. (**a**) Time-of-flight secondary ion mass spectroscopy (ToF-SIMS) depth-profile of relative elements of fresh and aged PSC devices with 3D (left) and 3D/2D (right) under 80 °C for 12 h. (**b**) Capacitance change of the devices based on 3D and 2D (n-butylamine (n-BA) and n-butyl ammonium iodide (n-BAI)) under thermal stress at 85 °C for 20 h. (**c**) Illustration of general ion migration in a 3D-based device (top) and suppressed ion migration in a 3D/2D-based device (bottom). (**d**) Illustrations of the inhibited escape of amine gas (MA) ions in 3D/2D perovskite (left) and MA volatilization in 3D perovskite (right) under thermal stress. Reprinted with permission from Ref. [44] Copyright 2017 WILEY–VCH for (**a**) and (**c**). Reprinted with permission from Ref. [36] Copyright 2018 American Chemical Society for (**b**) and (**d**).

The decomposition of a 3D perovskite structure is usually expected under constant thermal stress, which triggers ion migration and makes the escaped ions diffuse towards the top electrode, damaging both the hole transport material (HTM) and top electrode [60]. Notably, the 2D capping layer has a great impact on not only the moisture tolerance, but also the thermal stress resistance. The thermal effect on the ion diffusion across the perovskite film was investigated by using the time-of-flight secondary ion mass spectrometry (ToF-SIMS) depth-profile, as shown in Figure 9a. After heating the 3D-based PSC at 80 °C for 12 h, the depth-profile of the iodide was notably different from the fresh sample, indicating the pronounced iodide intensity (about 100 times greater) at the interface between the Ag electrode and PCBM [44]. The iodide diffusion toward the interface imposes the high possibility of the formation of AgI and thus implies degradation of the 3D film [60]. On the other hand, BAI-treated 3D/2D-based PSC showed a negligible difference in the iodide depth-profile, regardless of heat treatment. In addition, the increased capacitance in the low-frequency regime was observed for the 3D device after being heated for 20 h at 85 °C, which was ascribed to ion migration by thermal stress [61,62], while the 3D/2D-based device suppressed the increase in capacitance at a low frequency (Figure 9b) [36]. As illustrated in Figure 9c, thermal-induced ion migration, iodide in particular, toward the top electrode is effectively suppressed by bulky cations for the 2D layer, such as BA and PEA [44]. Besides, it has been reported that MA vacancies and the high density of iodine derived from MA volatilization are major causes of the degradation of 3D perovskite [60]. The volatilization of MA is inhibited in the 3D/2D bilayered structure due to the presence of a bulky cation in the 2D capping layer, which not only acts as a barrier to prevent the ion migration, but also makes it difficult for the bulky cation to replace the MA/FA vacancy due to the size difference (Figure 9d) [36].

**Figure 10:**
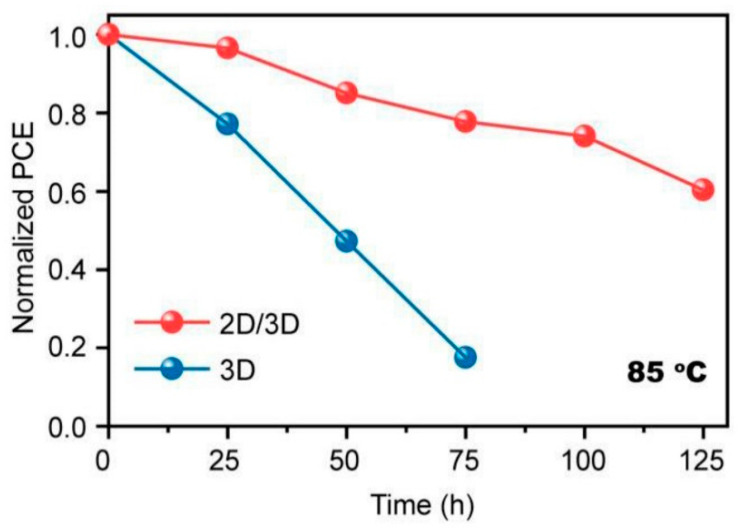
Thermal stability of devices based on 3D and 3D/2D bilayered perovskite films. Reprinted with permission from Ref. [45] Copyright 2020 American Chemical Society.

Figure 10 shows the normalized PCE of devices at 85 °C [45]. The device based on a 3D/2D bilayered structure apparently demonstrated an improved thermal stability compared to the device with a 3D film. The XRD intensity of PbI_2_, formed by thermal stress, was higher for the 3D film, though a PbI_2_ peak appeared for both 3D and 3D/2D films [45]. Furthermore, the thermal stability was highly governed by the thickness of the 2D capping layer in the 3D/2D bilayered structure [28]. A thicker 2D capping layer with 8 mg/mL PEAI enabled the device to retain 90% of the initial PCE for over 100 h at 60 °C. The device with 1 mg/mL PEAI, however, maintained 75% of the initial PCE at the same condition [28].

### 5.2. Enhanced Photovoltaic Performance

#### 5.2.1. Suppressed Charge Recombination

The most prominent feature enabled by the 2D capping layer is the surface passivation effect, which is directly responsible for the suppressed charge recombination. The growth of a 2D layer on the surface of a 3D film not only passivates the defect by acting as an ion scavenger during the formation of the capping layer [35], but also inhibits the ions from migrating toward the adjacent layers during the device operation [36,44]. The suppressed charge recombination is frequently monitored by an increased PL intensity, with a prolonged carrier life time in the presence of the 2D capping layer compared to the pristine 3D film [25,26,28,29,30,31,32,33,34,36,37,39,40,41,45]. The PL intensity corresponding to the 3D perovskite film was considerably enhanced by post-treatment with n-BAI [41]. Similarly, the increased PL intensity with BAI was further enhanced by replacing BAI with HAI owing to the low interfacial defects with HAI [37]. Time-resolved photoluminescence (TR-PL) results were indeed in accordance with the tendency of steady-state PL, depending on the 2D capping layer. When FEA^+^ contributed to the 2D in the bilayered structure, the carrier lifetime in the perovskite layer increased two-fold [25]. In the case of post-treatment with mixed salts (FA:PEA = 1:1), the average lifetime was also increased from 172 ns for the control 3D film to 314 ns for the mixed salts [33]. The charge recombination behavior was also studied by using impedance spectroscopy, where the addition of a 2D capping layer resulted in an increased charge recombination resistance [25,28,32,45]. Furthermore, the reduced ideality factor with DABr close to 1 [30] and the increased recombination time with PEAI [44] are indicative of the passivated traps induced by the formation of a 2D layer. The overall observation of suppressed non-radiative recombination is readily reflected in the improved photovoltaic performance, particularly in *V*_OC_ [39,63,64,65,66], which is well-summarized in Table 1.

#### 5.2.2. Energy Level Alignment

The effect of a 2D capping layer on the work function of perovskite film was investigated by measuring the contact potential difference (CPD) between a Pt-coated probe and the perovskite samples. Kelvin probe force microscopy (KPFM) was used to record the CPD with and without the 2D capping layer based on post-treatment with PEAI [28]. The 3D/2D bilayered structure with PEAI demonstrated −108 mV of CPD, while the pristine 3D film showed −41 mV of CPD, leading to an increase in *V*_OC_ [28]. The downshift of the Fermi level was indeed beneficial for hole extraction, with well-matched energy alignment with HTM. Furthermore, energy band alignment of the 2D layer is favorable for charge extraction in the bilayered structure. The 2D capping layer has been frequently introduced into the normal structure, n-i-p, where the 2D layer is sandwiched between the 3D perovskite and HTM. Ultraviolet photoelectron spectroscopy confirmed that the valence band of the 2D layer was located slightly higher than that of 3D, being favorable for hole extraction [32,42]. On the other hand, the conduction band of the 2D layer was aligned considerably higher than that of the 3D film, owing to the higher bandgap of 2D [32,42]. Therefore, the photogenerated electrons in the conduction band of 3D perovskite were blocked by the 2D layer acting as an energy barrier adjacent to HTM, which effectively restrained the interfacial charge recombination between the electrons in the conduction band of 3D and the holes in the valence band of HTM.

#### 5.2.3. Relieved I-V Hysteresis

Conventional layered perovskite solar cells demonstrate severe I-V hysteresis due to the large exciton binding energy and the inefficient charge extraction of 2D perovskite materials [17,18,67]. On the other hand, a highly relieved I-V hysteresis is generally monitored from the 3D/2D bilayered perovskite-based device [28,29,32,33,35,37,38,39,41,44,45,46]. The passivation effect by the growth of 2D capping layer was found to deactivate existing traps or lower the trap density of the 3D film, leading to the reduced I-V hysteresis in parallel to the enhanced PL intensity and *V*_OC_ in the 3D/2D bilayered structure [39,68,69,70]. The enhanced crystallinity of underlying 3D phase with the improved charge extraction of 3D/2D bilayered structure was indeed beneficial for the I-V hysteresis reduction [68,69]. Furthermore, the 2D capping layer acts as a barrier for the ion migration [36,44], being responsible for the suppressed I-V hysteresis [70].

## 6. Perspective

The commercial use of PSCs has been consistently challenged by a low device stability. Along with the evolution of encapsulation technology for PSCs to avoid any extrinsic parameter affecting the device’s stability, intensive research on materials and device structures has been carried out, with the aim of improving the device’s intrinsic stability. Recently, the 3D/2D bilayered perovskite structure has become an effective strategy for obtaining high-efficiency PSCs with a remarkable stability. The growth of low-dimensional perovskite on the photoactive 3D film enables the 3D/2D bilayered structure to be strongly resistant to various stress, leading to an outstanding long-term stability. Furthermore, remarkably suppressed charge recombination with efficient charge extraction in the 3D/2D structure leads to an improved photovoltaic performance. Therefore, the achievement of both efficiency and stability by the aid of the 3D/2D bilayered structure makes the PSCs more promising technology for commercial demands. The development of a new moiety in the organic cation for the 2D layer would bring a beneficial feature, while a delicate tuning of the interface would strengthen the effect of the 3D/2D bilayered structure not only in photovoltaics, but also in optoelectronics.

## Figures and Tables

**Table 1:** Organic salts employed for 2D layer formation.

	Chemical	Molecular Structure	Method	3D			3D/2D Bilayered		Ref
				Composition	*V*_OC_ (V)	PCE (%)	*V*_OC_ (V)	PCE (%)	
Alkyl-	n-Butylammonium iodide (n-BAI)	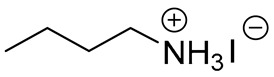	In-situ growth (solvent: IPA)	Cs_0.05_(MA_0.17_FA_0.83_)Pb(I_0.83_ Br_0.17_)_3_	0.955	14.17	1.05	15.74	[31]
				FAPbI_3_	1.06	19.02	1.11	19.50	[33]
				Cs_0.07_Rb_0.03_FA_0.765_MA_0.135_PbI_2.55_Br_0.45_	1.16 (R)	20.02 (R)	1.20 (R)	22.77 (R)	[41]
				MAPbI_3_	1.08	17.75	1.09	18.85	[36]
			Vacuum deposition (thermal evaporator)	MAPbI_3_	1.01	17.30	1.02	16.50	[46]
			Vapor-assisted (sealed box)	MAPbI_3_	1.14	18.98	1.16	19.48	[45]
	n-Butylamine (n-BA)	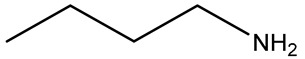	In-situ growth (solvent: CB)	MAPbI_3_	1.08	17.75	1.11	19.56	[36]
	Ethanediamineiodide (EDAI)	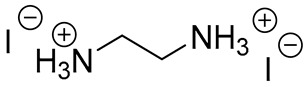	In-situ growth (solvent: IPA)	FAPbI_3_	1.04 (R) 0.94 (F)	16.23 (R) 13.08 (F)	1.09 (R) 1.04 (F)	17.96 (R) 16.88 (F)	[29]
	Cyclopropylammonium iodide (CAI)	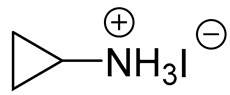		MAPbI_x_Cl_3__−x_	0.92	13.12	0.92	13.86	[24]
	Hexylammonium iodide (HAI)	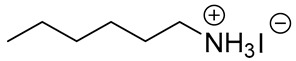		Cs_0.07_FA_0.79_MA_0.14_Pb(I_0.88_Br_0.12_)_3_	1.10	18.83	1.14	20.62	[37]
	Octylammonium iodide (OAI)	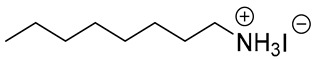		Cs_0.05_(MA_0.17_FA_0.83_)Pb(I_0.83_ Br_0.17_)_3_	0.955	14.17	1.022	15.19	[31]
	Diethylammonium bromide (DABr)	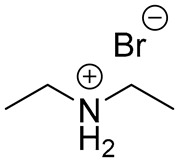		MAPbI_3_	1.04	14.37	1.06	18.30	[30]
	iso-Butylammonium iodide (i-BAI)	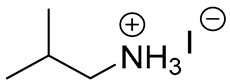		(FAPbI_3_)_0.85_(MAPbBr_3_)_0.15_	0.895		0.995	21.7	[39]
	5-ammoniumvalericacid iodide (5-AVAI or AVAI)	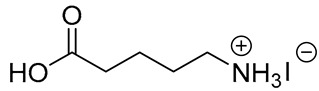		MAPbI_3_	1.01	17.3	1.06	18.0	[26]
				(FAPbI_3_)_0.88_(CsPbBr_3_)_0.12_	0.986 (R)	13.2(R)	1.06 (R)	16.03 (R)	[32]
Phenyl-	Phenylethylammonium iodide (PEAI)	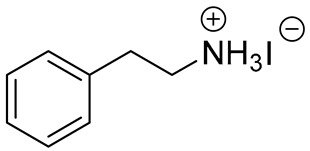	In-situ growth (solvent: IPA)	Cs_0.05_(MA_0.17_FA_0.83_)_0.95_Pb(I_0.83_ Br_0.17_)_3_	1.05	17.02	1.11	18.51	[28]
				FAPbI_3_	1.06	19.02	1.14	21.15	[33]
			Anti-solvent (solvent: toluene)	MAPbI_3_	1.10	18.75	1.17	19.89	[44]
			Vacuum deposition (thermal evaporator)	MAPbI_3_	1.056 (R)	18.9 (R)	1.061(R)	17.7 (R)	[47]
	Benzylamine (BA)	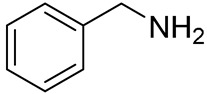	In-situ growth (solvent: CB)	Cs_0.15_FA_0.85_Pb(I_0.73_Br_0.27_)_3_	1.16	12.9	1.24	17.1	[42]
Fluoro-	Pentafluorophenylethylammonium iodide (FEAI)	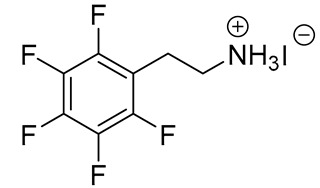	In-situ growth (solvent: IPA, immersing)	Cs_0.04_FA_0.92_MA_0.04_PbI_3_	1.045	20.02	1.096	22.16	[25]
	4,4,4-trifluorobutylammonium iodide (FBAI)	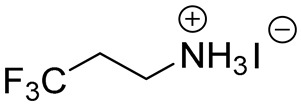	In-situ growth (solvent: IPA)	FAPbI_3_	1.06	19.02	1.07	17.5	[33]
	4-fluorophenyl-ethylammonium iodide (FPEAI)	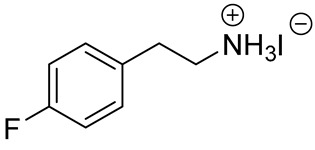			1.06	19.02	1.06	17.14	
				Cs_0.1_FA_0.77_MA_0.13_PbI_2.59_Br_0.41_	1.104	19.48	1.127	20.53	[40]
	6,6,6-trifluoro-4-oxo-5,5-bis(trifluoromethyl)hexan-1-aminium iodide (A43)	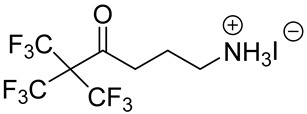		Cs_0.1_FA_0.74_MA_0.13_PbI_2.48_Br_0.39_	1.112	18.78	1.126	20.00	[34]
			Single precursor solution	MA_0.9_FA_0.1_PbI_3_	1.04	17.98	1.11	20.13	
Thiophene-	2-thiophenemethylammonium iodide (2-TMAI)	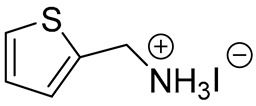	In-situ growth (solvent: IPA)	[(FAPbI_3_)_0.87_(MAPbBr_3_)_0.13_]_0.92_(CsPbI_3_)_0.08_	1.124 (1.041 *)	20.48 (19.01 *)	1.132 (1.049 *)	19.97 (17.91 *)	[35]
	3-thiophenemethylammonium iodide (3-TMAI)	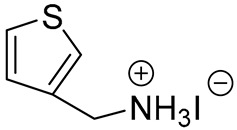					1.132 (1.032 *)	20.59 (18.55 *)	
	2-thiopheneethylammonium iodide (2-TEAI)	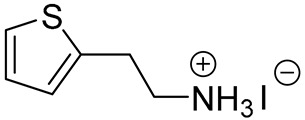					1.117 (1.010 *)	19.42 (15.70 *)	

R, reverse scan; F, forward scan; and *, value as prepared.
